# ESUR recommendations for MR imaging of the sonographically indeterminate adnexal mass: an update

**DOI:** 10.1007/s00330-016-4600-3

**Published:** 2016-10-21

**Authors:** Rosemarie Forstner, Isabelle Thomassin-Naggara, Teresa Margarida Cunha, Karen Kinkel, Gabriele Masselli, Rahel Kubik-Huch, John A. Spencer, Andrea Rockall

**Affiliations:** 1 Department of Radiology, Landeskliniken Salzburg, Paracelsus Medical University, Müllner Hauptstr. 48, A-5020 Salzburg, Austria; 2 Sorbonne Universités, UPMC Univ. Paris 06, Institut Universitaire de Cancérologie, Assistance Publique – Hôpitaux de Paris (AP-HP), Hôpital Tenon, Service de Radiologie, 54 avenue Gambetta, 75020 Paris, France; 3 Serviço de Radiologia, Instituto Portugues de Oncologia de Lisboa Francisco Gentil, R. Prof. Lima Basto, 1099-023 Lisboa, Portugal; 4 Institut de Radiologie, Clinique des Grangettes, Chemin des Grangettes 7, CH 1224 Chêne-Bougeries, Switzerland; 5 Radiology Department, Sapienza University, Viale del Policlinico 155, 00161 Rome, Italy; 6 Institut of Radiology, Departement of Medical Services, Kantonsspital Baden, Im Ergel, CH-5404 Baden, Switzerland; 7 Department of Radiology, St James’s University Hospital, Beckett Street, Leeds, LS9 7TF UK; 8 Consultant Radiologist, The Royal Marsden Hospital NHS Foundation Trust, London, UK; 9 Visiting Professor, Imperial College, London, UK

**Keywords:** Magnetic resonance imaging, Ovarian neoplasm, Recommendations, Diagnostic imaging, Ovarian cancer

## Abstract

**Abstract:**

An update of the 2010 published ESUR recommendations of MRI of the sonographically indeterminate adnexal mass integrating functional techniques is provided. An algorithmic approach using sagittal T2 and a set of transaxial T1 and T2WI allows categorization of adnexal masses in one of the following three types according to its predominant signal characteristics. T1 'bright' masses due to fat or blood content can be simply and effectively determined using a combination of T1W, T2W and FST1W imaging. When there is concern for a solid component within such a mass, it requires additional assessment as for a complex cystic or cystic-solid mass. For low T2 solid adnexal masses, DWI is now recommended. Such masses with low DWI signal on high b value image (e.g. > b 1000 s/mm^2^) can be regarded as benign. Any other solid adnexal mass, displaying intermediate or high DWI signal, requires further assessment by contrast-enhanced (CE)T1W imaging, ideally with DCE MR, where a type 3 curve is highly predictive of malignancy. For complex cystic or cystic-solid masses, both DWI and CET1W—preferably DCE MRI—is recommended. Characteristic enhancement curves of solid components can discriminate between lesions that are highly likely malignant and highly likely benign.

***Key Points*:**

• *MRI is a useful complementary imaging technique for assessing sonographically indeterminate masses.*

• *Categorization allows confident diagnosis in the majority of adnexal masses.*

• *Type 3 contrast enhancement curve is a strong indicator of malignancy.*

• *In sonographically indeterminate masses, complementary MRI assists in triaging patient management.*

**Electronic supplementary material:**

The online version of this article (doi:10.1007/s00330-016-4600-3) contains supplementary material, which is available to authorized users.

## Introduction

The previous guidelines for MR imaging of the sonographically indeterminate adnexal mass suggested a basic examination involving T1-weighted imaging (T1WI) and T2-weighted imaging (T2WI) to determine the nature and key signal characteristics of the mass, supplemented by additional oblique T2W imaging, fat-suppressed T1W (FST1W) or contrast-enhanced T1W (CET1W) imaging, depending on the key characteristic of the mass [1].

Recently, much effort has been invested in improving pre-surgical diagnosis of adnexal tumours by developing risk models and scoring systems using sonography [2–4]. In clinical routine, 5–25 % of adnexal lesions will remain indeterminate after sonography [2]. Even using the International Ovarian Tumour Analysis group (IOTA) simple rules, 22 % of lesions remained indeterminate on ultrasound (US) [4]. Most of these turn out to be common benign entities such as haemorrhagic lesions, fat-poor mature teratomas, uterine leiomyomas and ovarian fibromas [5]. The clinical impact of defining whether an indeterminate mass is benign or malignant is enormous. Women believed to have ovarian cancer may require radical cytoreductive surgery by a specialist surgeon in gynaecological oncology [6–8]. Furthermore, women with suspected malignancy may require transfer to a specialist institution. Conversely, benign adnexal masses may either be managed conservatively or undergo simple resection by a general gynaecologist.

In addition, with the increasing use of pelvic MRI, adnexal masses may also be identified as an incidentaloma. In our original guidelines, we suggested that where radiologist supervision of the examination was possible, an algorithmic approach could be used in order to tailor the examination, and that in most cases an accurate diagnosis could be achieved using one or two ‘problem-solving’ sequences in addition to the compulsory sequences. We have reconsidered these guidelines in light of recent clinical and imaging developments, and present our updated recommendations in Table 1.

**Table 1:** MR imaging protocol (2016)

Patient preparation	Intravenous smooth muscle relaxant Placement of intravenous cannula
Basic MR sequences	Sagittal T2W of the pelvis Pair of T1W, T2W through the indeterminate mass ±T2W sequence in the long axis of the uterus^1^
Problem-solving sequences	T1 ‘bright’ mass—FST1W T2 ‘dark’ solid mass (site of origin)—oblique T2W^a^ T2 ‘dark’ solid mass (nature)—DWI^b^ T2 solid mass—DWI^b,c^ and CET1W^d^ Cystic-solid mass—DWI^b,c^ and CET1W^d^

*Note*: Modifications to the previous recommendations are highlighted in grey

*FST1W* fat-suppressed T1-weighted, *DWI* diffusion-weighted imaging, *DCET1W* dynamic contrast- enhanced ﻿T1-weighted

^a^In many cases, this oblique T2W sequence along the long axis of the uterus (‘ovarian axis’) suffices. In other cases, a plane selected across the maximum point of contact of the mass and uterus is required to determine whether it is ovarian or uterine in origin and to look for bridging vessels

^b^A solid mass which has low signal on DWI sequences with b values of ≥ 800 s/mm^2^ can be regarded as benign, and CET1W imaging is unnecessary

^c^As T2 solid masses with intermediate to high DWI signal may be benign or malignant, additional CET1W imaging is required

^d^Ideally, with DCE MRI, where a type 3 curve is highly predictive of malignancy

## New imaging techniques

### Diffusion-weighted imaging (DWI)

There are now several studies confirming that DWI has a valuable role in MR imaging of the adnexal mass [9–14].

**Fig. 1:**
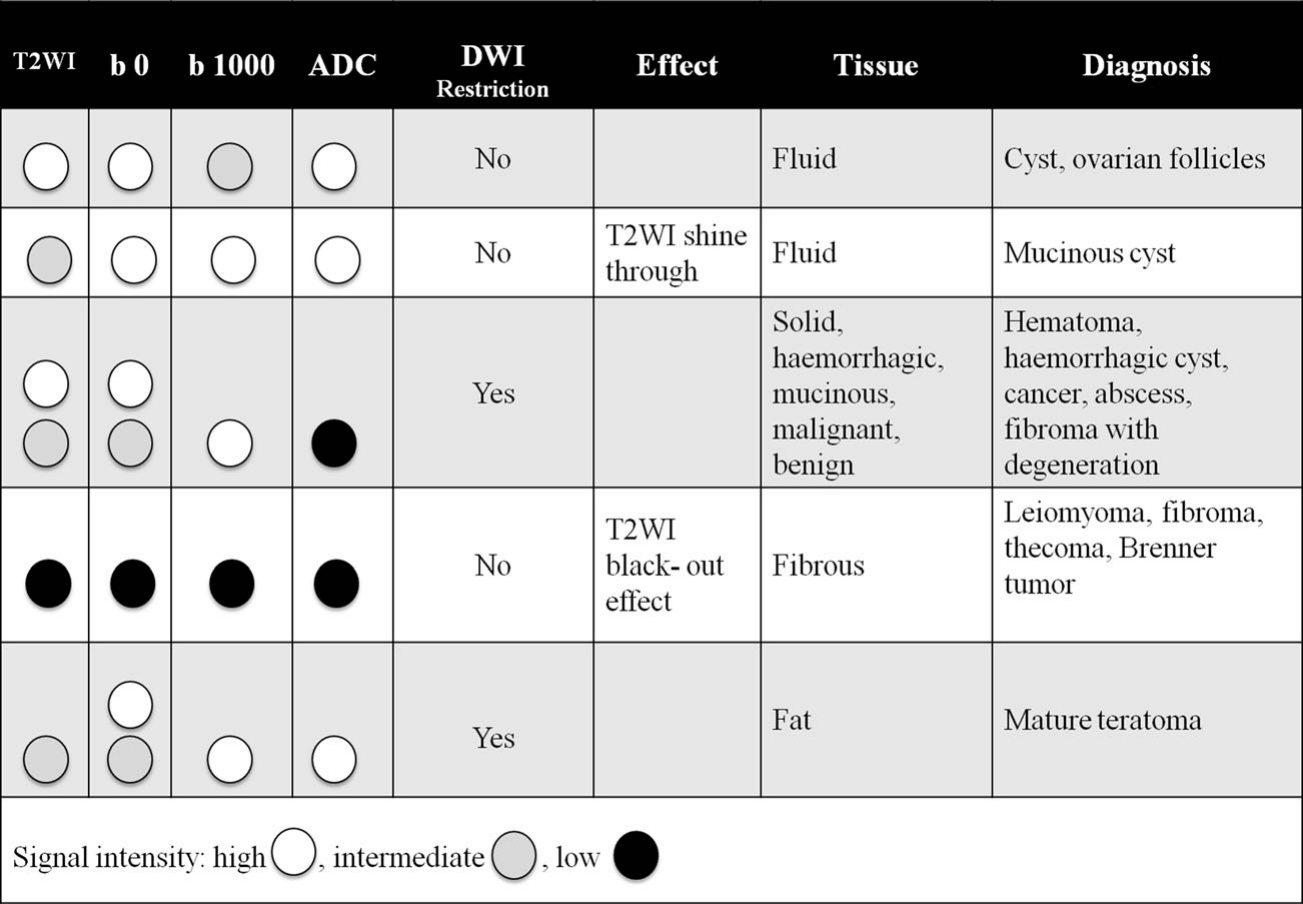
Characterization of adnexal masses by combining T2WI and DWI

The correct DWI technique is ensured by using a high enough b value to suppress any high signal intensity (SI) from freely diffusing water molecules, whilst keeping sufficient signal-to-noise ratio to identify pathology that has restricted water diffusion. For the female pelvis, we use the urine in the bladder as internal reference to guarantee that the chosen high b value is satisfactory. The urine is high in SI at b0, and decreases as the b value increases. When the bladder SI is fully suppressed, the optimal b value for adnexal mass characterization is achieved. For gynaecological imaging characterization, the optimal b value is usually 800–1000 s/mm^2^, but may be increased up to 1200 or 1400 s/mm^2^ [10]. Once the DWI sequence has been optimized, the lesion can then be evaluated (Table 2 and Fig. 1).

**Table 2:** How to integrate DWI in diagnostic algorithm

Diagnostic steps	DWI signal at high b value	Background	Diagnosis
1. Check urine in bladder	Urine remains high in SI	Need to increase the high b value	Cannot evaluate adnexal mass DWI
	Very low SI	Adequate high b value	Can now evaluate adnexal mass DWI
2. Characteristics of T2WI and DWI in adnexal mass	Low T2WI SI Low DWI SI	Highly likely benign	Fibroma Cystadenofibroma Benign solid component
	Any T2WI SI Residual signal on DWI	Non-specific	Mature teratoma Endometrioma Cancer Metastases
3.Compare DWI with ADC SI	High SI on high b and high SI on ADC	T2WI shine through	May be seen in cysts
	High SI on high b value and low ADC	Restricted diffusion—non-specific	Mature teratoma Endometrioma Cancer
4. ADC measurement	Characterization of lesion is not possible based on ADC quantification	Cancer tissue has low ADC but this is non-specific	Overlap between benign and malignant lesions

*SI* signal intensity, *DWI* diffusion-weighted imaging, ADC apparent diffusion coefficient, *T2WI* T2-weighted imaging

The key points for the interpretation of DWI are as follows:

- DWI SI of water (i.e. urine in the bladder) is dark.
- The DWI SI of the mass must be compared with that on T2WI and ADC.
- Due to considerable overlap, ADC quantification is not useful for assessing adnexal masses [13]. In view of this, there is—up to now—no indication to perform multi-b value diffusion.

**Fig. 2:**
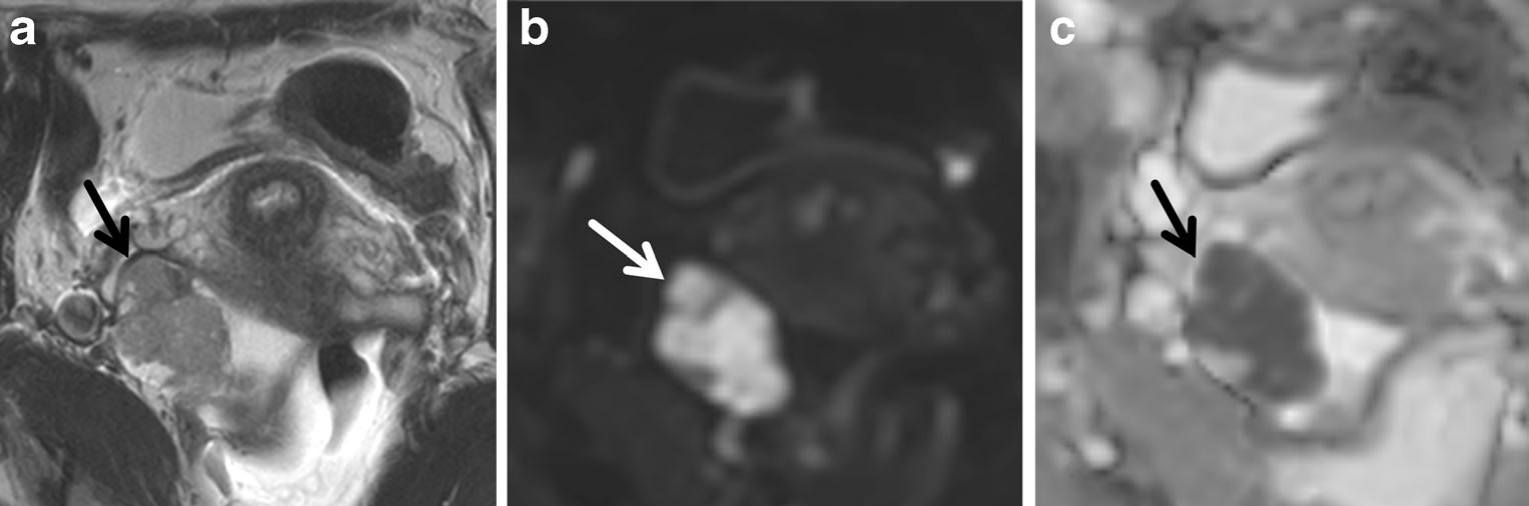
Ovarian carcinoma confined to the right ovary (*arrow*) displaying intermediate SI on T2WI and restricted diffusion characterized by high SI on the high-b-value (b1200) image and loss of signal on ADC

The presence of ‘diffusion restriction’ is evidenced by high DWI SI on high-b-value images with corresponding low SI on the ADC map (Fig. 2). This differs from those tissues in which the water molecules are not highly restricted, where the DWI SI may be low on the high b value and high on ADC or, in the presence of very high T2 SI lesions, the lesion may be high and high (T2 shine-through effect).

Initial studies evaluating DWI in adnexal masses reported high SI in mature cystic teratomas and endometriomas as well as in malignant masses, whilst the majority of fibromas and other benign masses had low DWI signal [9, 14]. These authors rightly cautioned against using DWI as a ‘standalone’ technique, due to the overlap in common benign and malignant masses [9]. DWI should not be applied in the diagnosis of mature ovarian teratomas and endometriomas. In practice, the great majority of these lesions can be accurately diagnosed by simply observing their T2W features and the characteristics on T1W and FST1W imaging.

Rather, the added value of DWI is in assessing non-fatty, non-haemorrhagic pelvic masses that are entirely solid, or complex masses that are either septate cysts or complex solid and cystic masses. Diagnostic confidence is increased by about 15 % when DW images are added to conventional images [12]. If the solid component of an indeterminate adnexal mass is of low SI on T2WI, and the entire mass displays low signal on DWI obtained with a b value of 800–1000 s/mm^2^, there is a very high likelihood of benignity [11].

DWI is thus diagnostic in the majority of predominantly solid benign adnexal masses, such as ovarian fibroma or cystadenofibroma, and in most pedunculated uterine leiomyomas. Moreover, a low T2W solid mass with low DWI signal is highly likely to be benign, irrespective of its pattern of contrast enhancement [12].

In these circumstances, DWI can thus replace CET1W MRI as a confirmatory sequence for benignity of a solid or partly solid indeterminate mass. This is of particular relevance in pregnant women in whom contrast administration is contraindicated but complementary imaging to US is warranted.

Conversely, when an indeterminate adnexal mass is solid or has a solid component with high signal on the high-b-value DWI, it may be benign or malignant, and CET1W MRI should be performed.

### Dynamic contrast-enhanced (DCE) MR imaging

Two European centres have done pioneering work on DCE MRI of complex adnexal lesions [15–20]. It is well known that factors related to tumour biological processes, such as VEGFR-2 expression and pericyte coverage index (PCI), are related to maximum uptake of gadolinium by the tumour [18].

Using semi-quantitative multiphase contrast-enhanced MRI, the predominant finding by both groups was that in adnexal masses where solid components demonstrate a rapid rate and high level of enhancement, there is a very high likelihood of malignancy, whereas a slow rate and low level of enhancement is associated with a high likelihood of a benign lesion [15–17, 20].

**Fig. 3:**
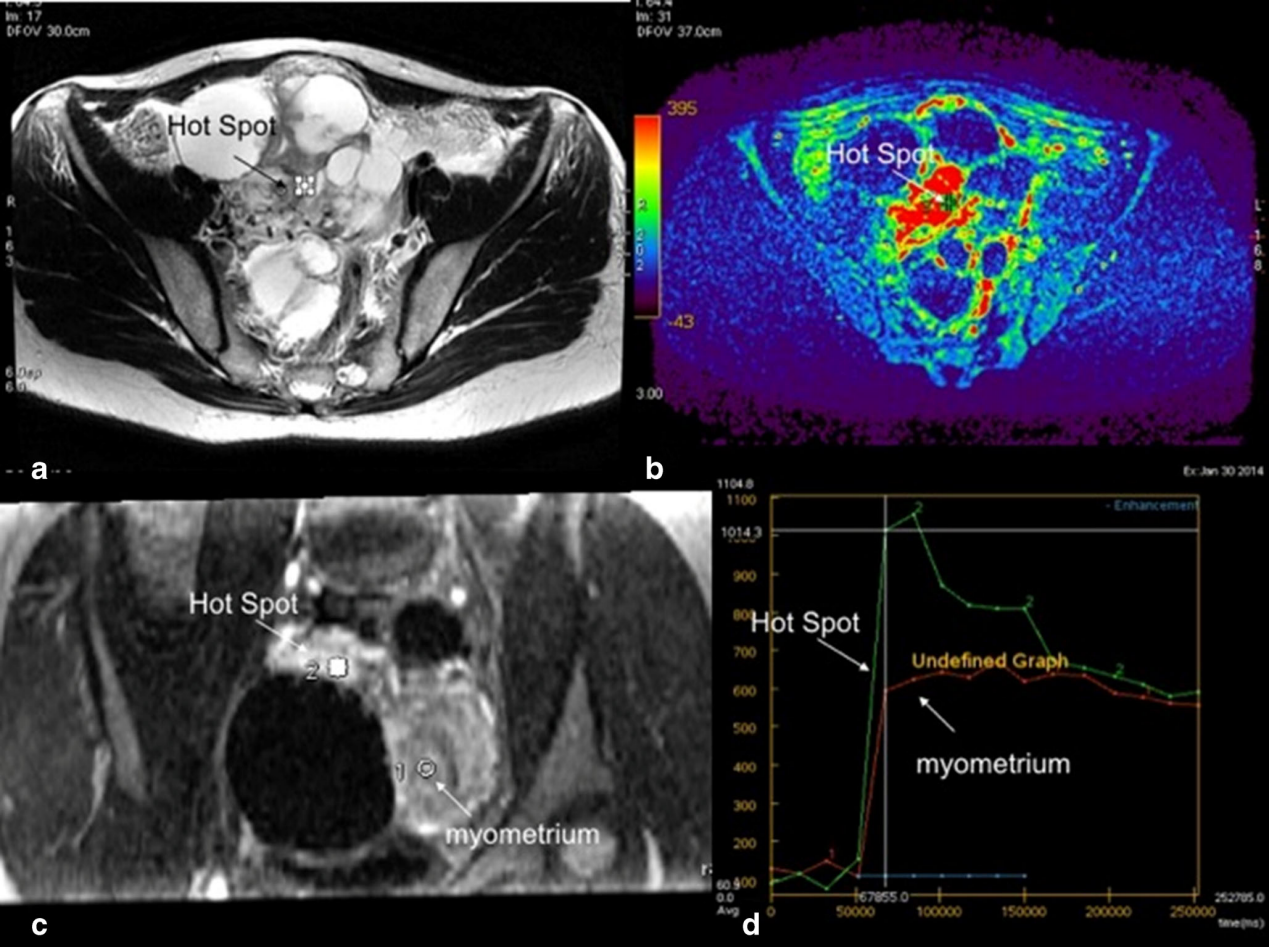
Technical assessment of DCE MR imaging in complex adnexal masses. This example shows a complex right ovarian mass with a solid component in intermediate T2W signal (**a**) that heterogeneously enhances after gadolinium injection. Parametric map (maximal slope) helps to determine the most suspicious location (hot spot) where the region of interest should be placed to build the time–intensity curve (**b**). To compare this curve with the myometrial curve, 3D T1W sequence must be reformatted in the coronal plane to place the two ROI (solid component and external myometrium) (**c**). Comparison of time–intensity curves shows that the solid component enhances according to a time–intensity curve type 3 (curve steeper than that of myometrium)

The analysis of dynamic contrast enhancement is based on the comparison of the time–intensity curve of the solid component in an adnexal mass with that of the external myometrium which serves as internal reference. Thus, a DCE MR sequence must be acquired in a plane that involves the solid component of the adnexal mass (i.e. solid papillary projections, thickened irregular septa or solid portion) and the myometrium (Fig. 3). Either the plane is selected by the radiologist and a 2D T1W sequence is performed, or better, a 3D T1W sequence may be acquired. Technical details for optimizing DCE MRI (Fig. 3) are provided in the technical appendix.

## Review process

The ESUR guidelines for MRI of sonographically indeterminate adnexal masses were published in 2010. In 2015, a re-evaluation of the current practice was initiated by the expert members. Questionnaires analysing recent clinical practice in imaging of sonographically indeterminate adnexal masses, notably the integration of DWI and DCE imaging, and the type of clinical protocol were collected from 13 European institutions and one centre in Japan. In addition, the literature published between 1999 and 2015 was reviewed. In two consensus meetings in 2015, a draft of the current update was developed and discussed, and was ultimately approved after distribution among the subcommittee members.

## Updates

### Indications for MRI in a sonographically indeterminate mass

The complementary use of MRI is most beneficial in the following clinical scenarios:

- A complex adnexal mass with equivocal malignant features
- A large pelvic mass of indeterminate origin
- A mass adjacent to the uterus with equivocal origin
- A solid adnexal mass

### Patient preparation

There are no new data. It is recommended that a smooth muscle relaxant is administered intravenously or intramuscularly.

### Diagnostic algorithm (Fig. 4a–c)

**Fig. 4:**
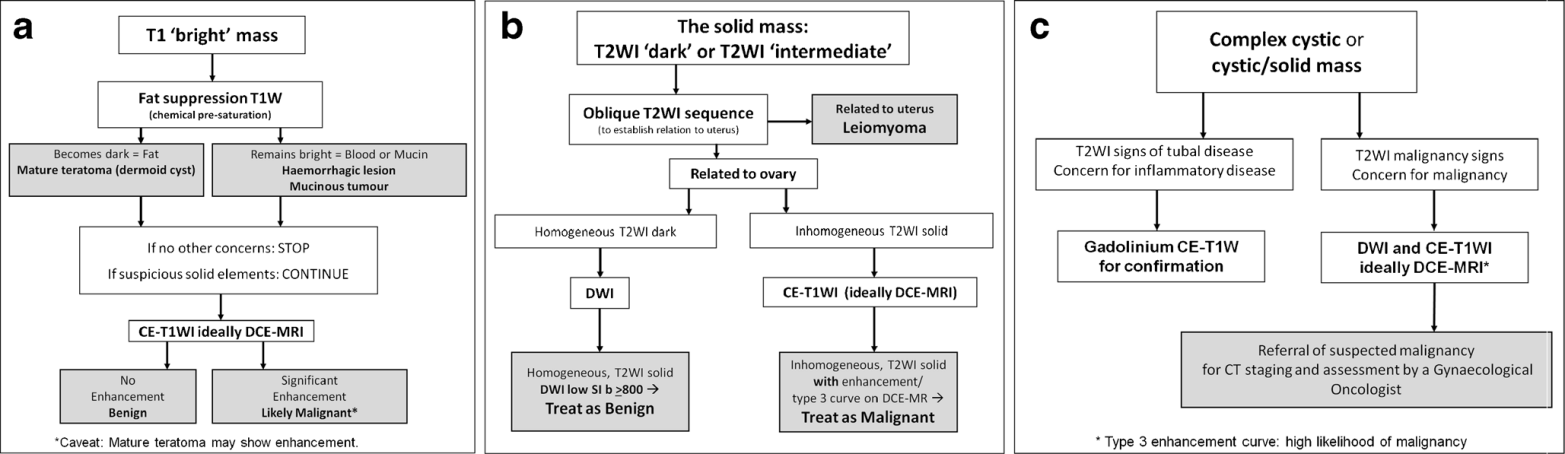
Flow charts with revised algorithm for T1 ‘bright’ masses (**a**), T2 solid masses (**b**), and complex cystic or cystic-solid masses (**c**)

There are no new data regarding this, and the recommendation remains that it requires basic and problem-solving imaging sequences. The basic imaging technique, as a minimum, comprises the following:

- A T2W sagittal sequence of the pelvis
- A pair of T1W and T2W sequences covering the adnexal mass and its relationship to the uterus in the same orthogonal (axial or coronal or oblique) plane with identical slice thickness

The choice of which plane is used is at the discretion of the supervising radiologist. The key is the identical position of the pair of T1W and T2W and—if performed—DWI and DCE/CET1W images to allow direct comparison of the entire mass.

For the 2010 guidelines [1], the decision tree divided indeterminate masses into three groups on the basis of their key characteristic on the basic T1W and T2W sequences. For the purposes of that algorithm, solid material had SI similar to muscle on T1-weighted sequences, and cyst contents had SI on T2W sequences similar to the urinary bladder. The three categories of mass were as follows:

- T1 ‘bright’ masses containing T1 high SI
- T2 solid masses with predominant signal either similar to skeletal muscle (T2 ‘dark’ solid masses) or higher than muscle (T2 ‘intermediate’ or mixed-signal solid masses)
- Complex cystic or cystic-solid masses

## T1 ‘bright’ masses (Fig. 4a)

There are no new data to guide assessment of such masses. These masses require FST1W imaging using chemical pre-saturation to distinguish fat from blood. The FST1W sequence should be performed in exactly the same plane as the T1W sequence to allow direct comparison.

Whilst haemorrhagic masses may have low signal on T2WI, it is their T1W ‘bright’ characteristic—a reflection of T1 shortening from extracellular methaemoglobin—that distinguishes them.

When there is concern for a solid nodule within a T1 bright mass, additional assessment as for a complex cystic or cystic-solid mass is required. An enhancing nodule within an endometrial cyst is a finding suggesting endometriosis-associated cancer [21]. It is recommended that the post-contrast appearance is reviewed on subtracted images to improve reporting accuracy.

As mature cystic teratomas rarely undergo malignant change, all portions of T1 bright masses should be carefully analysed for signs of such transformation as capsular breach or a large heterogeneous solid component.

Caution is warranted regarding application of DWI and DCE and overreliance on their findings for assessment of T1 bright masses. Both may yield positive findings with benign cystic teratomas. Epidermoid components of teratomas show diffusion restriction similar to malignant lesions, and components of benign teratomas may also rarely rapidly enhance or show type 3 time–intensity curves [9, 16, 22–24]. DWI of haemorrhagic lesions may have a confusing appearance, and offers no additional diagnostic value [10].

Our recommendation is for no change in the evaluation of T1 bright masses. Their fat or blood content can be determined simply and effectively using a combination of T1W, T2W and FST1W imaging. However, if these masses have solid aspects, or if a teratoma displays a large heterogeneous solid component, further assessment with gadolinium injection is advised.

## T2 solid masses (Fig. 4b)

Well-delineated, sonographically indeterminate solid adnexal masses raise concerns for ovarian metastases, yet in practice almost all of these will turn out to be benign fibrous or fibromuscular masses such as uterine leiomyomas or ovarian fibromas [5].

**Fig. 5:**
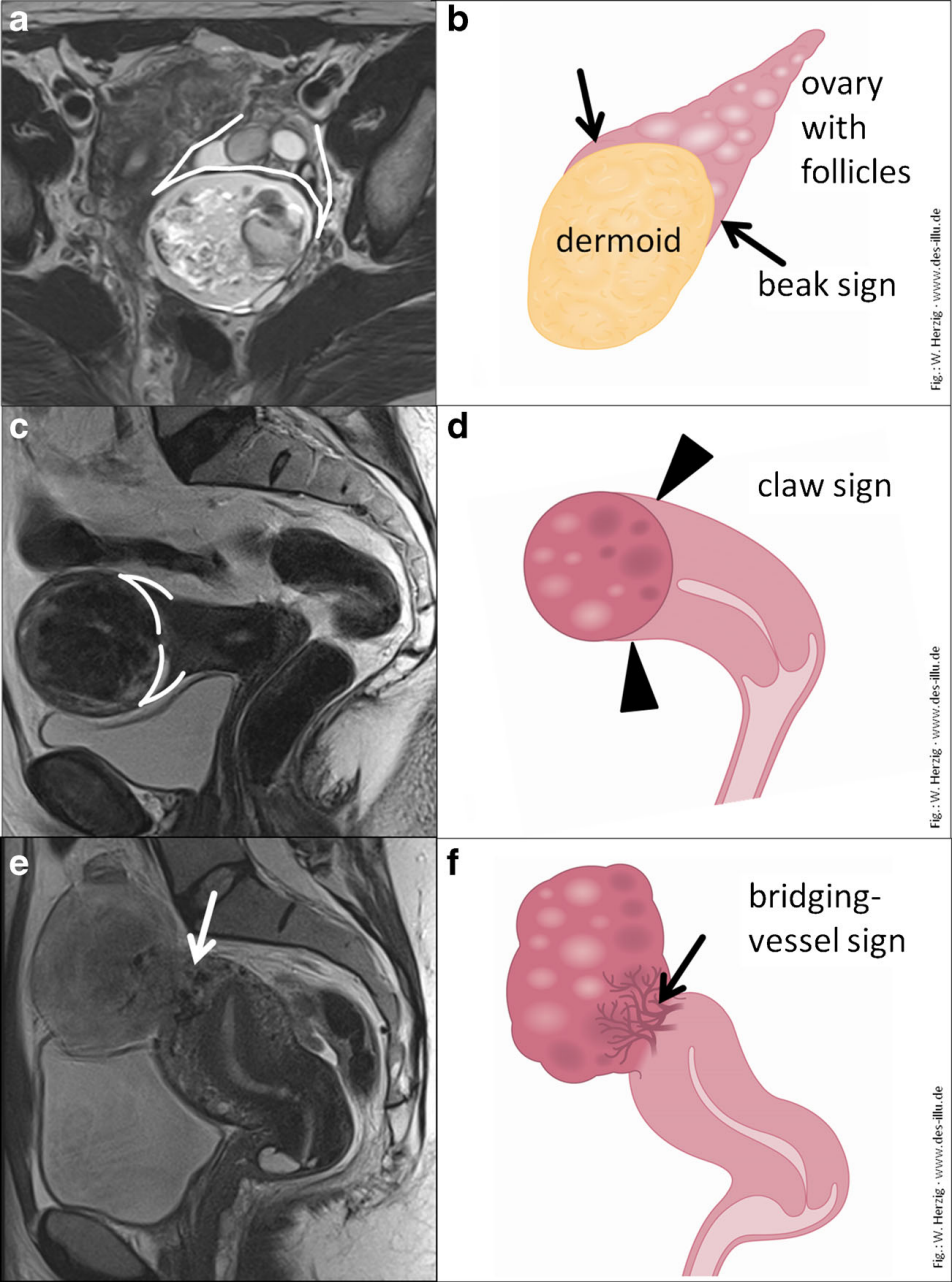
Differentiation of ovarian versus uterine origin. Beak sign indicating ovarian origin in a benign teratoma (*arrows* and *outlined* in **a** ﻿and **b**). The most important differential diagnosis of a solid adnexal mass includes uterine leiomyoma, which can be differentiated by the claw sign (*arrow* and *outlined* in **c** and **d**) or in broad-based leiomyomas by bridging vessels (*arrow* in **e** and **f**)

The first consideration is defining their anatomic site of origin, ovarian or uterine (Fig. 5). An ovarian fibroma is separate from the uterus, and often only the contralateral normal ovary is seen. In a uterine leiomyoma, there may be normal uterine tissue draped around the solid mass, holding it like a ‘claw’ (Fig. 5c and d). Uterine leiomyoma may also be attached to the uterus by a stalk which contains the ‘bridging vessels’ that supply it (Fig. 5e and f). The conspicuity of this pedicle and the bridging vessels is made more obvious using an oblique T2W sequence through the maximum point of contact between the mass and the uterus.

From feedback through personal communication, we are aware that in clinical practice, many radiologists feel more comfortable confirming the diagnosis of ovarian fibroma with CET1W imaging. Ovarian fibroma is typically slowly and minimally enhancing, and displays a type 1 curve on DCE MRI [14, 25]. DCE MRI may also be useful in differentiating pedunculated subserosal leiomyomas from ovarian fibromas. Studies have shown that enhancement of pedunculated subserosal leiomyomas parallel those of the adjacent myometrium, whereas contrast uptake in fibromas is more delayed [14, 25].

We now recommend DWI in T2W low-signal solid ovarian adnexal masses, which are most likely to be an ovarian fibroma or a Brenner tumour. Such a solid mass having entirely low signal on DWI sequences with high b values can be regarded as benign, and CET1W imaging is unnecessary.

It is our recommendation that a solid adnexal mass with T2 intermediate SI or a T2 dark mass showing other than low DWI signal be further assessed by CET1W imaging, ideally with DCE MRI when this is available.

## Complex cystic or cystic-solid masses (Fig. 4c)

In our previous guidelines [1], we recommended CET1W imaging for assessment of masses which raised concerns for malignancy: some solid masses (as discussed above), solid components within cystic masses, and nodular or irregular thickening of internal septa or of the inner or outer aspects of the wall of a mass. CET1W imaging remains the benchmark technique to look for malignant features, and is the one most widely available [26].

However, both DWI and DCE MRI, when available, are recommended as adjunct investigations. Persistent high signal using b values > 800 s/mm^2^, with corresponding low ADC signal indicating diffusion restriction, is found in ovarian cancer (Fig. 2). However, several benign lesions, including benign cystic teratomas, endometrial cysts, some fibrothecomas, degenerating leiomyomas, and Brenner tumours, may also display such signal characteristics on DWI. Furthermore, ADC quantification shows too much overlap to confidently allow prediction of malignancy. Conversely, low DWI signal using a high b value is highly predictive of a benign lesion [11]. Recent data underscore the value of including DCE in the routine work-up of indeterminate adnexal masses. A retrospective analysis in 87 women with complex adnexal masses demonstrated a correct change in 16–24 % of lesion characterization when both DWI and DCE were used [12]. In addition to peritoneal implants, the presence of a time–intensity curve type 3 is the best predictor of malignancy, and this enhancement pattern was found in no benign but in 58 % of malignant tumours [17]. Of note, the benign sclerosing stromal tumour of the ovary may display early contrast enhancement; however, the centripetal enhancement pattern may suggest a specific diagnosis in this extremely rare tumour among women of childbearing age [27].

A time–intensity curve type 1, a weak and progressive enhancement after gadolinium injection, predicts benignity and may be especially helpful in recognizing benign stromal tumours and cystadenofibroma that may display a high DW signal [14, 28].

Our recommendations for complex cystic or cystic-solid masses are that DWI and DCE MRI be used, if available, as adjuncts to CET1W imaging.

Tubo-ovarian inflammatory disease commonly causes complex cystic masses, with more indolent or chronic forms typically presenting as sonographically indeterminate masses. Complex folds and other mural abnormalities within tubal disease may mimic neoplastic features [29]. Caution is warranted in using DWI, as the high signal in these masses is produced mainly by the liquid purulent component and not the solid aspect. Thus, DWI must be carefully analysed in combination with T1W and T2W images. CET1W imaging can increase the conspicuity and improve the diagnosis of tubal disease, and may provide some clues as to disease activity and to complications such as abscess formation.

## New developments

An MRI scoring system based on standard T1WI and T2WI appearances supplemented by DWI and DCE MRI features—the ADNEX MR scoring system—has been proposed [17]. A prospective multicentre study is being conducted in conjunction with the female pelvic imaging working group of the ESUR (EURAD-MR Classification) in order to analyse the potential impact of this model on therapeutic strategy and to test its reproducibility. First results are expected in 2016.

18-FDG PET/CT can provide additional information to transvaginal ultrasound (TVUS) in the differential diagnosis of benign from malignant pelvic lesions. However, in the assessment of sonographically indeterminate lesions, it is currently not recommended, due to its adherent limitations, including physiological uptake in normal ovaries, uptake in common benign lesions, and its potential lack of uptake in cystic or in necrotic tumours. Furthermore, with reported sensitivity of 52–58 % and specificity of 76–78 % for characterization of ovarian masses, it is inferior to MRI [30]. Increased uptake of FDG in an ovarian mass in women of post-menopausal age is indicative of a malignant tumour [30]. However, caution is warranted in benign teratomas in both pre-and postmenopausal women [31].

The added value of the integration of PET/MRI for characterization of ovarian lesions has yet to be validated [32].

## Summary of new recommendations

An algorithmic MRI approach using basic and problem-solving sequences under radiologist supervision will ensure a specific diagnosis in the vast majority of sonographically indeterminate adnexal masses.

We do not recommend any change in the evaluation of T1 ‘bright’ masses (Fig. 4a). Their fat or blood content can be determined simply and effectively using a combination of T1W, T2W and FST1W imaging. When there is concern for a solid nodule within such a mass, it requires additional assessment as for a complex cystic or cystic-solid mass.

We now recommend that DWI be applied for low T2 solid adnexal masses (Fig. 4b). Such masses with low DWI signal can be regarded as benign. Any solid adnexal mass which shows intermediate or high DWI signal requires further assessment by CET1W imaging, ideally with DCE MRI. This technique may also be useful in the differentiation of uterine from ovarian origin in such a mass.

We now recommend that for complex cystic or cystic-solid masses, both DWI and DCE MRI are used, if available (Fig. 4c). Otherwise, such masses are appropriately examined using CET1W imaging.

Our recommendations are shown in new algorithms and summarized in Table 1. These ESUR guidelines now supersede those from 2010.

## Electronic supplementary material

Below is the link to the electronic supplementary material.

ESM 1 (DOCX 14 kb)
