# Supplementary material for: ESUR recommendations for MR imaging of the sonographically indeterminate adnexal mass: an update
Source: Eur Radiol. 2016 Oct 21;27(6):2248–57. doi: 10.1007/s00330-016-4600-3 (PMC5408043; doi:10.1007/s00330-016-4600-3)
Supplement: Supplementary file 1 — (DOCX 14 kb) [file 330_2016_4600_MOESM1_ESM.docx]

**Technical addendum:**

Technique and requirements to perform optimal DCE MRI

1. The imaging volume should be acquired as 3D isotropic voxels, in order to allow reformatting in any plane with a maximal slice thickness of 3mm.
2. DCE acquisition is ideally done in the axial plane, in line with the T2 and DWI for ease of cross correlation of the SI characteristics.
3. In order to ensure enough data points to form enhancement curves, a minimum time resolution of 15 seconds per acquisition should be performed. In order to achieve this time resolution, it may be that fat saturation may not be possible depending on the system. Subtraction will allow the suppression of any pre-contrast high T1 signal intensity, whether the DCE is acquired with or without fat saturation. The acquisition should start prior to contrast injection (we suggest at least 30 seconds prior to contrast injection) and should continue for at least 3 minutes.
4. Curve analysis technique [12]:
   1. Ensure the time intensity curve is evaluated using percentage or relative enhancement (not absolute enhancement)
   2. Identify the most rapid/highest level enhancement in the adnexal mass for selection of ROI for solid tissue enhancement curve. Colour coded maps facilitate the identification of the most suspicious spot within the solid portion of the lesion (figure 3)
   3. Once the most rapid area of enhancement is identified in the mass, the chosen adnexal ROI is positioned in the same plane as the myometrial ROI in order to ensure a fair comparison with the myometrial enhancement curve. This may require an oblique reformat.
   4. Place ROI on the outer myometrium.
   5. Curve type 1: Gradual increase in signal intensity of solid tissue without a well-defined shoulder.
   6. Curve type 2: A moderate initial increase in the signal intensity of solid tissue relative to that of myometrium, followed by a plateau. Curve type 3: An initial increase in the signal intensity of solid tissue that is steeper than that of myometrium.
